# Supplementary material for: Optimized flow cytometric detection of transient receptor potential vanilloid-1 (TRPV1) in human hematological malignancies
Source: Med Oncol. 2022 Apr 28;39(6):81. doi: 10.1007/s12032-022-01678-z (PMC9046313; doi:10.1007/s12032-022-01678-z)
Supplement: Supplementary file 1 — Supplementary file1 (PDF 103 kb) [file 12032_2022_1678_MOESM1_ESM.pdf]

Supplementary Fig.S1: Overview of the step-by-step Flow cytometry protocol for the detection of TRPV1 in human leukocytes in peripheral blood

Material:

Material	Supplier	Catalogue#
5 mL polypropylene tubes	Falcon®	352008
Anti-TRPV1	LifeSpan Biosciences	LS-C150735
Isotype control	Santa Cruz Biotechnology	sc-3888
Goat anti-rabbit IgG-FITC	Santa Cruz Biotechnology	sc-2012
BD FACS™ lysing solution	BD biosciences	349202
Fix/perm kit	BD Cytofix/Cytoperm™	554714

Blocking solution: 10% human blood group AB serum, supplemented with 1% bovine serum albumin (BSA), 0.05% sodium azide, and made in the permeabilization reagent.

Protocol:

1. Label 5 mL polystyrene Falcon® tubes according to the experiment design, including isotype control and secondary antibody only tubes.
2. Add the fluorescently-conjugated surface antibodies (according to suppliers' instructions or antibody titration studies) carefully to the bottom of the tube.
3. After performing a complete blood count on the human blood sample, aliquot 1 x 10^6 leukocytes to the sample and control tubes. Vortex gently to mix.
4. Incubate the tubes at room temperature in the dark, for 10 min.
5. Add 2 ml of the RBCs lysis buffer to the blood samples, vortex gently to mix. Incubate at room temperature in the dark for 5-10 min. **Note:** This incubation must **NOT** exceed 10 minutes, or leukocytes will start lysing.
6. Centrifuge for 5 min at 300- 350xg in a swing-bucket centrifuge, then remove supernatant. Resuspend with 3 mL cold PBS and centrifuge again and discard supernatant.
7. Add 250 µL/ tube of BD fixing reagent (Cytofix/Cytoperm™), mix gently by pipetting. Incubate for 20 min at 4°C. Wash 2X in the permeabilization reagent at 1ml/tube/wash.
8. Add 50 µL of the blocking solution. Incubate at room temperature for 15 min in the dark.
9. Add 0.5 µg anti-TRPV1 primary antibody, or 0.5 µg (1/25) isotype control, mix gently. Incubate at 4°C for 45 min in the dark.
10. Wash samples thrice with BD permeabilization reagent (1ml/tube/wash). Centrifuge for 3 min at 350xg and remove supernatant.
11. Resuspend cells in 50 µL of secondary Ab, (1/25, diluted in the permeabilization reagent), and mix gently. Incubate for 20 min, at 4°C in the dark.
12. Wash samples thrice with BD permeabilization reagent (1ml/tube/wash). Centrifuge for 3 min at 350xg and remove supernatant.
13. Resuspend the cells in Phosphate Buffered Saline (Ca^{2+} and Mg^{2+} free), and run on the flow cytometer.
