# Supplementary material for: Optimized flow cytometric detection of transient receptor potential vanilloid-1 (TRPV1) in human hematological malignancies
Source: Med Oncol. 2022 Apr 28;39(6):81. doi: 10.1007/s12032-022-01678-z (PMC9046313; doi:10.1007/s12032-022-01678-z)
Supplement: Supplementary file 2 — Supplementary file2 (PDF 298 kb) [file 12032_2022_1678_MOESM2_ESM.pdf]

Supplementary 2

Table S1. General characteristics of patients with hematological malignancies

Age	Years (range)	31- 85
Sex	Male	28
	Female	21
Subjects	*De novo*	12
	Under treatment	37
	Control	21
Diagnosis	B-NHL	28
	MM	12
	Acute Monocytic Leukemia	2
	MPD	4
	HCL	1
	ALL	1
	PTCL/NOS	1

MM: multiple myeloma; B-NHL: B- Cell Non-Hodgkin's lymphoma; MPD: Myeloproliferative Disorder; HCL: Hairy-Cell Leukemia; ALL: Acute Lymphoblastic Leukemia; PTCL-NOS: Peripheral T-cell lymphoma/not otherwise specified.

Table S2. Comparison of mean TRPV1 MFI in THP-1, U266B1 and U937 cell lines and normal leukocytes

Cell line	n	Mean MFI (10^5) (Cell Line)	Mean MFI (10^5) (Normal Leukocytes)	Ratio	Change
**THP-1**	4	2.38	0.97	2.5	Increased
**U266B1**	1	1.81	0.76	2.38	Increased
**U937**	1	1.19	0.76	1.57	Unchanged

MFI: Median Fluorescence Intensity; n= number of experiments.

Table S3. Characteristics of MM patients compared to control group subjects used for analysis of TRPV1 expression by flow cytometry

		Patients				Control Subjects		TRPV1 Expression
**Status**	**IDN**	**Age range** (Years)	**Sex**	**MFI** (10^5)	**CRP** (mg/L)	**IDN**	**MFI** (10^5)	**MFI Ratio**
De novo	P09	82	M	1.13	4	C05	1.12	1.01
	P21	73	M	1.16	3	C10	1.55	0.75
	P35	72	F	3.57	5	C12	1.17	***3.05***
Undergoing Chemotherapy	P02	57	M	0.92	1	C01	1.04	0.88
	P04	67	F	0.86	1	C03	0.68	1.27
	P05	57	F	1.20	1	C06	1.31	0.92
	P06	69	F	1.08	3	C06	1.33	0.81
	P07	68	F	0.89	2	C06	1.31	0.68
	P08	71	M	1.11	3	C04	1.28	0.86
	P26	66	M	2.03	NA	C10	1.68	1.20
	P28	58	M	1.62	1	C14	1.24	1.31
	P39	73	F	2.62	7	C15	1.00	***2.62***

IDN: patient/control identification number (P: patient, C: control); MFI: Median Fluorescence Intensity; CRP: C-reactive protein; NA: not available data; *: increase in TRPV1; M: male, F: female

Table S4. Characteristics of B-NHL patients compared to control subjects used for analysis of TRPV1 expression by flow cytometry

				Patients		Control Subjects		TRPV1 Expression
**Status**	**IDN**	**Age** (Years)	**Sex**	**MFI** (10^5)	**CRP** (mg/L)	**IDN**	**MFI** (10^5)	**MFI Ratio**
	P20	74	M	2.00	58	C10	1.76	1.14
De novo	P31	55	M	0.79	2	C14	1.23	0.64
	P33	62	F	1.58	2	C12	1.69	0.93
	P49	83	F	1.19	3	C21	1.44	0.83
	P03	80	F	0.95	8	C02	0.89	1.07
	P10	71	F	1.54	4	C07	1.32	1.17
	P11	70	F	1.69	1	C07	1.20	1.41
Undergoing	P12	31	M	1.87	NA	C09	1.30	1.44
Chemotherapy	P13	29	M	1.56	2	C09	1.29	1.21
	P14	41	M	1.36	4	C09	1.21	1.13
	P15	45	F	1.55	1	C09	1.17	1.33

P19	68	M	1.67	60	C09	1.22	1.37
P22	65	M	1.97	7	C10	1.71	1.15
P23	85	F	1.72	1	C11	1.43	1.20
P25	77	F	3.43	19	C11	1.39	***2.47***
P27	70	M	1.35	3	C13	0.97	1.40
P29	77	M	1.81	15	C13	0.93	1.94
P30	56	M	2.10	4	C14	1.21	1.73
P32	65	F	1.26	3	C12	1.73	0.73
P34	80	F	1.54	4	C12	1.65	0.94
P36	79	M	2.16	1	C16	2.38	0.91
P37	79	F	2.73	3	C15	1.00	***2.73***
P38	72	M	2.12	1	C17	2.38	0.89
P40	46	M	2.35	4	C17	2.28	1.03
P41	76	M	2.42	3	C16	2.14	1.13
P42	78	M	2.29	1	C16	2.13	1.08
P44	65	F	1.57	1	C19	2.04	0.77
P45	73	M	1.73	5	C18	1.37	1.26

Table S5: Characteristics of other haematological malignant cancers patients compared to control subjects used for analysis of TRPV1 expression by flow cytometry

					Patients		Control Subjects		TRPV1 Expression
**Status**	**IDN**	**Disease**	**Age range** (Years)	**Sex**	**MFI** (10^5)	**CRP** (mg/L)	**IDN**	**MFI** (10^5)	**MFI Ratio**
De novo	P16	MPD (CMML)	71	M	1.61	NA	C09	1.45	1.12
	P17	HCL	43	M	0.47	NA	C09	1.05	0.45
	P18	PTCL -NOS	63	M	1.63	106	C09	1.20	1.36
	P47	ALL	68	M	2.81	63	C20	1.60	1.76
	P48	AML (M4)	77	F	1.05	NA	C21	0.68	1.54
Undergoing Chemotherapy	P24	MPD	40	F	1.92	1	C11	1.57	1.22
	P43	MPD (ET)	64	F	1.98	1	C19	2.02	0.98
	P01	MPD (CML)	66	M	0.46	4	C03	0.38	1.21
	P46	AML (M4)	43	M	1.33	3	C18	1.25	1.06

IDN: patient/control identification number (P: patient, C: control); MFI: Median Fluorescence Intensity; CRP: C-reactive protein; NA: non available data; M: male, F: female; HCL: Hairy-Cell Leukaemia; PTCL-NOS: Peripheral T-cell lymphoma/ not otherwise specified; ALL: Acute Lymphocytic Leukaemia; AML (M4): Acute Myelomonocytic Leukaemia; MPD: Myeloproliferative Disorder; CML: chronic Myelocytic Leukaemia; CMML: Chronic Myelomonocytic Leukaemia; ET: Essential thrombocythaemia.
