# Supplementary material for: Optimized flow cytometric detection of transient receptor potential vanilloid-1 (TRPV1) in human hematological malignancies
Source: Med Oncol. 2022 Apr 28;39(6):81. doi: 10.1007/s12032-022-01678-z (PMC9046313; doi:10.1007/s12032-022-01678-z)

Original Fig 1C (upper section):

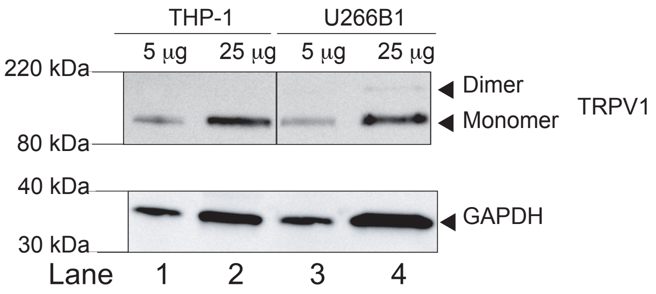

Fig 1C (upper section): raw WB data

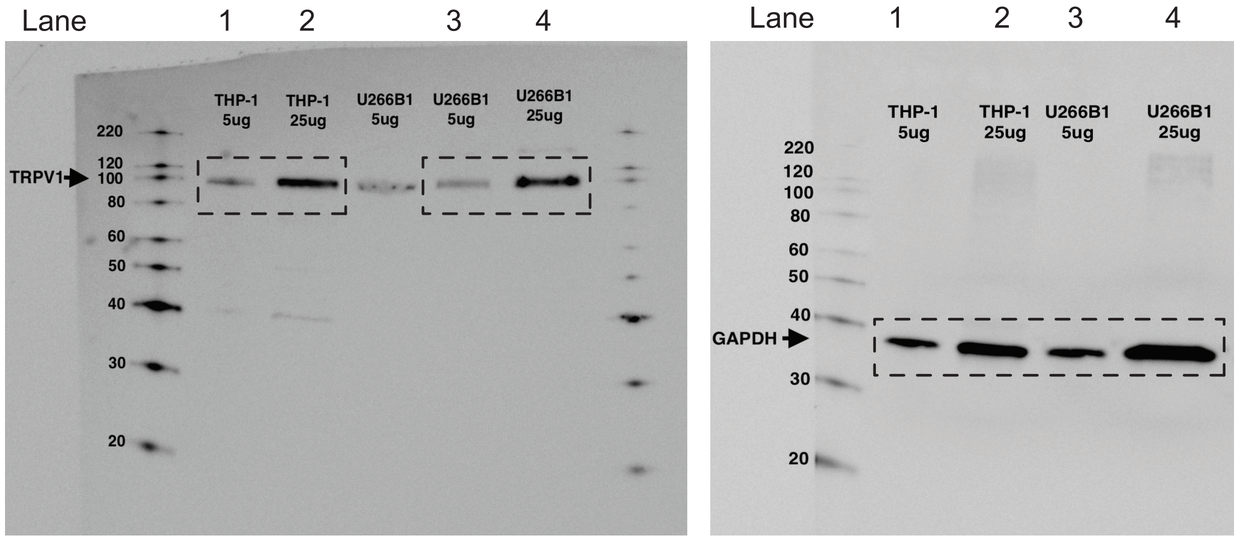

Original Fig 1C (lower section):

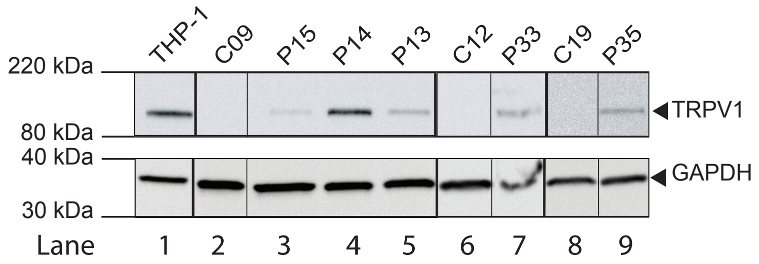

Fig 1C (lower section): raw WB data

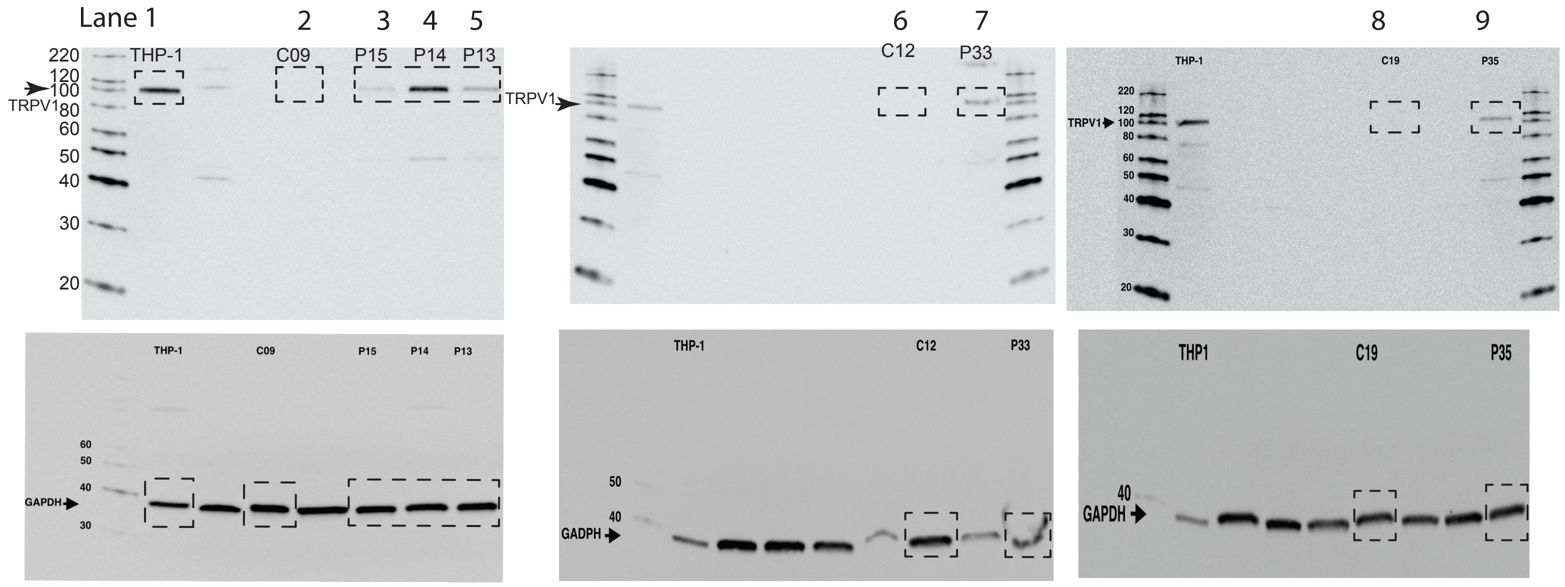

Supplement: Supplementary file 3 — Supplementary file3 (PDF 19833 kb) [file 12032_2022_1678_MOESM3_ESM.pdf]
